# Clonal Evolution through Loss of Chromosomes and Subsequent Polyploidization in Chondrosarcoma

**DOI:** 10.1371/journal.pone.0024977

**Published:** 2011-09-20

**Authors:** Linda Olsson, Kajsa Paulsson, Judith V. M. G. Bovée, Karolin H. Nord

**Affiliations:** 1 Department of Clinical Genetics, University and Regional Laboratories, Skåne University Hospital, Lund University, Lund, Sweden; 2 Department of Pathology, Leiden University Medical Center, Leiden, The Netherlands; National Cancer Center, Japan

## Abstract

Near-haploid chromosome numbers have been found in less than 1% of cytogenetically reported tumors, but seem to be more common in certain neoplasms including the malignant cartilage-producing tumor chondrosarcoma. By a literature survey of published karyotypes from chondrosarcomas we could confirm that loss of chromosomes resulting in hyperhaploid-hypodiploid cells is common and that these cells may polyploidize. Sixteen chondrosarcomas were investigated by single nucleotide polymorphism (SNP) array and the majority displayed SNP patterns indicative of a hyperhaploid-hypodiploid origin, with or without subsequent polyploidization. Except for chromosomes 5, 7, 19, 20 and 21, autosomal loss of heterozygosity was commonly found, resulting from chromosome loss and subsequent duplication of monosomic chromosomes giving rise to uniparental disomy. Additional gains, losses and rearrangements of genetic material, and even repeated rounds of polyploidization, may affect chondrosarcoma cells resulting in highly complex karyotypes. Loss of chromosomes and subsequent polyploidization was not restricted to a particular chondrosarcoma subtype and, although commonly found in chondrosarcoma, binucleated cells did not seem to be involved in these events.

## Introduction

Chondrosarcoma is the collective term for a group of bone tumors characterized by hyaline cartilage differentiation and otherwise heterogeneous morphological and clinical features [1]. Based on morphology four subtypes can be recognized. By far, the most common variant is conventional chondrosarcoma, which may originate from the medullar cavity (central) or the surface of the bone (peripheral) [2]. Less frequently encountered subtypes include dedifferentiated, mesenchymal and clear cell chondrosarcomas. When resectable, the prognosis is excellent. Inoperable or metastatic tumors are, however, generally lethal as there is currently no effective chemotherapy and the tumors are largely insensitive to radiotherapy. Histological grade is the best predictor of clinical course, but the discrimination between low-grade and high-grade lesions is influenced by variability among observers [3], [4]. Cytogenetic and molecular genetic analyses have shown that chondrosarcomas display chromosome numbers ranging from hyperhaploid to hyperhexaploid [5], [6]. Some tumors harbor related clones of two ploidy levels, suggesting that chondrosarcoma cells may polyploidize to the extent of duplicating their entire set of chromosomes, most probably trough a single event. We have previously identified genomic changes that support loss of chromosomes and subsequent polyploidization in neoplastic chondrocytes by using cytogenetics and array comparative genomic hybridization (CGH) [7]. However, as the two chromosomal homologues cannot be discriminated by these techniques it has not been possible to ascertain whether clones with more than 46 chromosomes arise through polyploidization of an originally hyperhaploid-hypodiploid clone or by other mechanisms. To determine genomic aberrations and mechanisms for aneuploidization in chondrosarcoma we have here used single nucleotide polymorphism (SNP) array analysis, which allows for the simultaneous detection of DNA copy number and loss of heterozygosity (LOH) pattern. We have also performed a literature survey of published karyotypes from chondrosarcomas. Furthermore, by combining immunofluorescence (IF) and fluorescence in situ hybridization (FISH) analyses we have evaluated if binucleated cells, which are commonly found in neoplastic cartilage [1], are involved in the polyploidization of chondrosarcoma cells.

## Results

### Published data on cytogenetic alterations in chondrosarcoma include loss of chromosomes and signs of polyploidization

**Figure 1:**
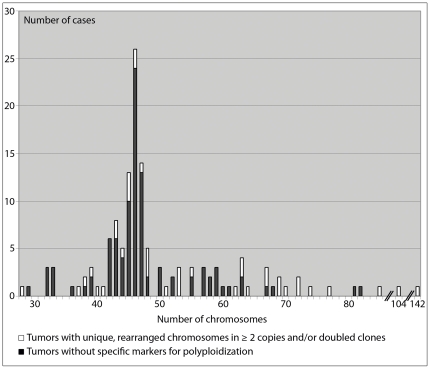
Ploidy levels of cytogenetically analyzed chondrosarcomas of bone. To date, 138 chondrosarcomas of bone with an aberrant karyotype (excluding myxoid chondrosarcomas and 45,X,-Y) have been published (Mitelman Database of Chromosome Aberrations and Gene Fusions in Cancer: http://cgap.nci.nih.gov/Chromosomes/Mitelman June 1, 2010). The ploidy levels vary from hyperhaploid to hyperhexaploid. In 39 of the cases rearranged chromosomes in at least two copies and/or a doubled clone were found, supporting polyploidization of chondrosarcoma cells.

A survey of published karyotypes from chondrosarcomas showed cytogenetic alterations (excluding 45,X,-Y) in 138 tumors (Fig. 1). Less than 46 chromosomes were found in 49 cases, 8 of which were hyperhaploid. In 9 of the hyperhaploid-hypodiploid tumors a corresponding doubled clone was detected. Of 89 tumors with 46 or more chromosomes, 27 cases displayed rearranged chromosomes in ≥2 copies and/or doubled clones. As rearranged chromosomes in ≥2 copies could result from duplication of all chromosomes their presence may indicate that the cells have polyploidized.

### Loss of heterozygosity is a common finding in chondrosarcoma

**Figure 2:**
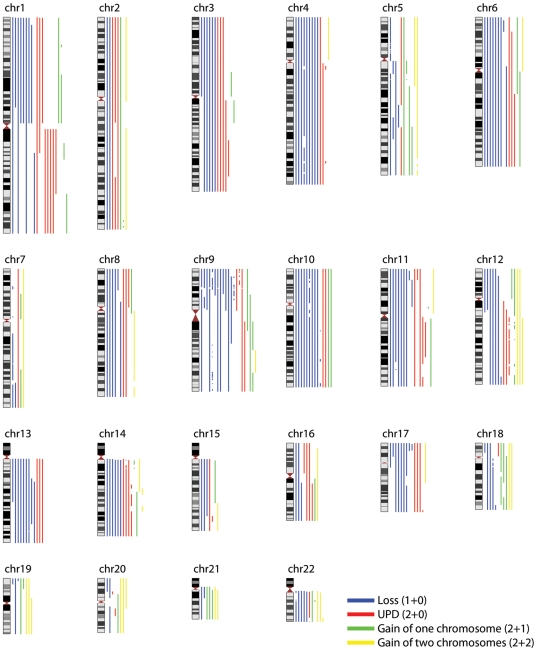
Genomic aberrations detected in 16 chondrosarcomas. Genomic loss, uniparental disomy (UPD) and gain of one or two copies identified by SNP array analysis are displayed for all autosomes. Loss of heterozygosity resulting from deletion or UPD was frequently found, except for chromosomes 5, 7, 19, 20 and 21. Detailed information on all aberrations affecting each individual case is shown in Figs. S1, S2, S3, S4. The imbalance map was created using the freely available software Genome Wide Viewer (http://www.well.ox.ac.uk/~jcazier/GWA_View.html).

Sixteen chondrosarcomas were investigated by SNP array analysis. Chromosomal aberrations were detected in all cases and the imbalances agreed well with previous array CGH and cytogenetic findings (Figs. S1, S2, S3, S4) [7]. In most cases, the majority of the autosomes displayed LOH resulting from loss of chromosomes or uniparental disomy (UPD) (Fig. 2).

### SNP array analysis reveals that chromosomes 5, 7, 19, 20 and 21 rarely show loss of heterozygosity

Chromosomes 5, 7, and 20 displayed regions which never were monosomic whereas chromosomes 19 and 21 were lost in only one case each (Fig. 2). The genomic regions 1.05–1.40 Mb, 7.56–8.07 Mb, 9.67–9.91 Mb, 22.39–22.85 Mb, 29.46–30.27 Mb, 32.12–32.92 Mb, 34.73–34.85 Mb, 34.87–35.15 Mb, 36.14–37.52 Mb, 37.63–38.07 Mb, 38.85–39.70 Mb, 40.23–41.47 Mb and 44.41–44.43 Mb in chromosome 5, pter-20.07 Mb, 23.23–54.74 Mb, 55.35–74.03 Mb, 108.16–109.18 Mb and 127.16–130.32 Mb in chromosome 7, and 22.43–32.37 Mb in chromosome 20 were not deleted in any case. Genomic material was lost from chromosomes 5, 7, 19, 20 and/or 21 in four to 13 of 138 published chondrosarcomas with aberrant karyotypes and in none to three of the eight published near-haploid chondrosarcomas, agreeing well with our SNP array results. Thus, chromosomes 5, 7, 19, 20 and 21 rarely displayed LOH and the vast majority of cytogenetically investigated chondrosarcomas showed at least two copies of these chromosomes.

### Genomic aberrations support polyploidization of chondrosarcoma cells

**Figure 3:**
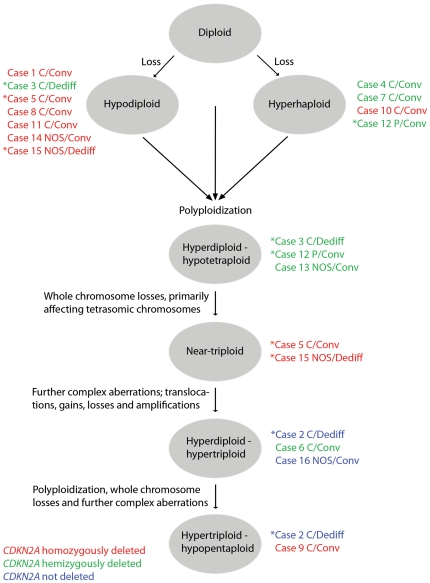
Inferred polyploidization events of hyperhaploid-hypodiploid chondrosarcoma cells affected by *CDKN2A* deletions. Polyploidization of hyperhaploid-hypodiploid chondrosarcoma cells was supported by the combined SNP array and cytogenetic findings. Hyperhaploid-hypodiploid clones were detected in eleven cases. In four of these and in five additional cases chromosome aberrations supporting polyploidization was found. Polyploidization may occur more than one time and the cells may be affected by further rearrangements, gains and losses of genetic material. In case 14, a hypodiploid chromosome number was detected and uniparental disomy was shown for several of the disomic chromosomes. The detected pattern of aberrations in this case suggested a potential developmental step with duplication of some monosomic chromosomes and did not support polyploidization of a hyperhaploid cell. In cases 2 and 16, the detected pattern of preserved heterozygosity for the vast majority of chromosomes indicates that the tumor cells never had been hyperhaploid or hypodiploid and that polyploidization affected diploid cells with both parental alleles retained. In these two cases *CDKN2A* was not affected by deletion, whereas in the remaining cases the gene was hemi- or homozygously lost. Cases in which clones of two ploidy levels were detected are indicated by asterisks.

Based on cytogenetic findings, the ploidy levels of the SNP array investigated tumors ranged from hyperhaploid to hypopentaploid (Fig. 3, Table S1). Cases 1, 4, 7, 8, 10 and 11 presented hyperhaploid-hypodiploid clones with retained heterozygosity for disomic chromosomes and structural rearrangements in single copies (Figs. S1, S2, S3, Table S1). Cases 3 and 12 showed hypodiploid and hyperhaploid clones, respectively, together with polyploid clones. SNP array cannot distinguish between the original and polyploidized clones but in both cases the SNP array results were in agreement with either or both of the karyotypic clones (Figs. S1 and S3, Table S1). Case 14 displayed a hypodiploid clone with mono- and disomic chromosomes (Fig. S4, Table S1). The majority of disomic chromosomes showed retained heterozygosity and four chromosomes displayed UPD. Structurally rearranged chromosomes were found in a single copy. Cases 6 and 13 showed hyperdiploid chromosome numbers, frequent LOH for disomic chromosomes and two maternal and two paternal alleles for the vast majority of tetrasomic chromosomes (Figs. S2 and S4, Table S1). Single copies of structurally rearranged chromosomes were detected in case 6 and in a minor clone in case 13. In cases 5 and 15, SNP array analysis detected hypodiploid cells whereas cytogenetic analysis showed cells with the doubled number of chromosomes as well as further changes (Figs. S2 and S4, Table S1). No rearranged chromosomes were found in case 5. In case 15, many chromosomal rearrangements were present in two copies. Case 9 was hypertriploid and displayed rearranged chromosomes in two or three copies, LOH for di- and trisomic chromosomes, and retained heterozygosity for genomic regions present in four to six copies (Fig. S3, Table S1). This combination of aberrations suggests that an original hypodiploid cell polyploidized more than one time (Fig. 3). In contrast to the other 14 cases, the vast majority of chromosomes in the near-triploid cases 2 and 16 displayed retained heterozygosity. This is most likely explained by polyploidization of diploid cells, followed by whole chromosome losses and acquisition of complex aberrations (Figs. S1 and S4, Table S1). In case 2, cytogenetic analysis also detected a related, hypopentaploid clone. The vast majority of rearranged chromosomes in cases 2 and 16 were present in single copies. To summarize, except for the two cases which seemed to have polyploidized from a diploid cell the majority of chondrosarcomas displayed SNP array results suggestive of a hyperhaploid-hypodiploid origin, with or without subsequent polyploidization (Fig. 3). After polyploidization the cells may be affected by further gains, losses and rearrangements of genetic material and polyploidization may be repeated.

### *CDKN2A* deletion is detected in tumors with a hyperhaploid-hypodiploid origin

The majority of the SNP array investigated chondrosarcomas were of the central conventional type, one case was a peripheral conventional chondrosarcoma and three were dedifferentiated chondrosarcomas (Fig. 3). The low number of peripheral conventional and dedifferentiated tumors precluded any comparison with regard to differences in chromosome number or type of aberration between the subtypes. There was no apparent difference in patient outcome between cases in which polyploidization was detected and cases in which only a hyperhaploid or hypodiploid clone was detected (Fig. 3, Table S1). *CDKN2A* was homozygously lost in eight cases and hemizygously deleted in six cases (Fig. 3, Figs. S1, S2, S3, S4). In these 14 cases, SNP patterns indicative of a hyperhaploid-hypodiploid origin, with or without subsequent polyploidization were detected.

### Binucleated chondrocytes do not seem to give rise to mononucleated polyploid cells

**Figure 4:**
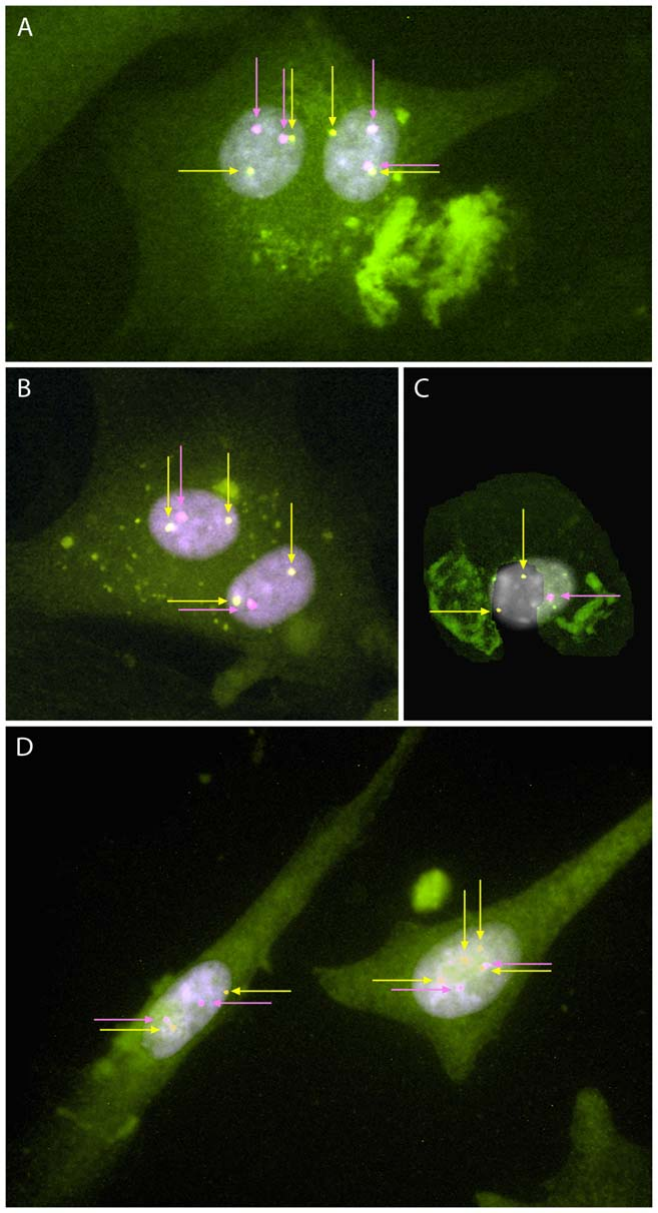
Ploidy levels of mono- and binucleated chondrocytes. Combined immunofluorescence and fluorescence in situ hybridization analyses were used to detect the ploidy levels of mono- and binucleated chondrocytes. To visualize individual cells a plasma membrane marker was detected by a fluorescently labeled antibody (green). Centromeric probes specific for chromosomes known to be monosomic (pink) and disomic (yellow) in hypodiploid cells and consequently disomic (pink) and tetrasomic (yellow) in hypertriploid cells were used for detection of ploidy levels. (A) Normal diploid nuclei as well as (B) hypodiploid nuclei are shown in binucleated cells (case 17). Mononuclear cells display (C) hypodiploid, (D) normal and hypertriploid nuclei (cases 18 and 15, respectively). The proportion of binucleated chondrocytes was 1–10% of all cells investigated with normal, hypodiploid and hypertriploid nuclei (Table S2). In binucleated cells both nuclei displayed the same ploidy level.

To investigate the involvement of binucleated chondrocytes in polyploidization, ploidy levels of both mono- and binucleated chondrocytes were examined in three chondrosarcomas as well as in normal cartilage. Combined IF and FISH analyses showed that binucleated chondrocytes represented 1–10% of the investigated cells, irrespective of their ploidy level, and that the two nuclei in binucleated cells consistently showed the same ploidy level (Fig. 4, Table S2). In chondrosarcomas, diploid, hypodiploid and hypertriploid nuclei were found. Cells with diploid nuclei likely represent normal cells present in the tumor sample. All nuclei of normal cartilage were diploid, excluding that the binucleated normal cartilage cells gave rise to mononucleated haploid or polyploid cells.

## Discussion

A chromosome number in the near-haploid range is a rare phenomenon in neoplasia, described in less than 1% of all cytogenetically reported tumors [5]. It has primarily been found in hematologic malignancies and in a few subtypes of solid tumors, including chondrosarcoma [5], [7], [8]. We here show that hyperhaploid-hypodiploid chondrosarcoma cells often polyploidize and subsequently may be affected by further gains, losses and rearrangements of genetic material and that polyploidization may occur more than one time. The majority of the autosomes frequently displayed LOH as a result of chromosome loss and, in some cases, subsequent duplication of the remaining homolog giving rise to UPD. It is obvious that loss of chromosomes is pathogenetically important in chondrosarcoma but bearing in mind the size of the lost regions it is not possible to speculate on the importance of individual genes for tumor development. Chromosomes 5, 7, 19, 20 and 21 nearly always displayed retained heterozygosity, which is in line with previously published cytogenetic information [5]. Why these particular chromosomes are retained and why the remaining autosomes are frequently lost remains elusive. It is notable that a similar pattern, albeit involving other chromosomes, is seen in hyperhaploid-hypodiploid acute lymphoblastic leukemia (ALL) [9], suggesting that retention of heterozygosity for certain chromosomes and loss of others may be a general phenomenon in tumors with less than 46 chromosomes. One possibility is that monoallelic gene expression is involved in the selection of lost and retained genomic regions in chondrosarcoma. The expression of about 1% of human genes is estimated to be affected by parental imprinting and these genes have been predicted by computational analysis to be present in all autosomes [10]. To date, imprinted genes have been confirmed in chromosomes 1, 6, 7, 11, 14, 15, 18 and 20. In addition, autosomal genes have been shown to display monoallelic expression due to random inactivation of maternal or paternal alleles, *i.e.* some cells express the maternal allele and other cells express the paternal allele; a pattern of expression that is maintained in descending cells [11]. The recurrent pattern of chromosome aberrations in chondrosarcoma may hence be attributed, at least partially, to allelic differences in gene expression.

It has previously been shown that polyploidization of chondrosarcoma cells can occur in peripheral chondrosarcoma [6], [8]. As the majority of the present cases were represented by central conventional and dedifferentiated chondrosarcomas it is evident that polyploidization of an original hyperhaploid-hypodiploid cell is not restricted to a particular subtype. Although based on an admittedly limited number of cases, there was no obvious difference in patient outcome between cases which displayed a sole hyperhaploid-hypodiploid clone and cases which also showed a duplicated clone. Thus, polyploidization is detected in all chondrosarcoma subtypes sufficiently analyzed to date and does not seem to be associated with progression of the disease.

Polyploidization has been demonstrated to occur during normal liver growth through cytokinesis failure in hepatocytes, giving rise to binucleated cells with diploid nuclei and subsequently to mononucleated tetraploid cells [12]. In chondrosarcoma, binucleated cells are commonly seen, but it is not known if these cells can give rise to mononucleated haploid or polyploid cells [1]. In the present study, the normal cartilage cells always displayed diploid nuclei. As there were no cells with non-diploid nuclei we conclude that binucleated normal chondrocytes did not give rise to mononucleated haploid or polyploid cells in our *in vitro* system. This could indicate that the same is true for chondrosarcoma cells and that polyploidization does not involve binucleated cells.

The concomitant detection of hyperhaploid-hypodiploid cells and the corresponding duplicated clones demonstrates that chondrosarcoma cells are initially affected by extensive loss of whole chromosomes followed by polyploidization. In some cells further genetic alterations occur. Using SNP array methodology, the previously unclear issue of how clones with more than 46 chromosomes form has thus been resolved (Fig. 3). Based on the present results and published cytogenetic information (Fig. 1), hyperhaploid chondrosarcomas do not seem to represent a separate group distinct from hypodiploid tumors. The mechanism behind the initial chromosome loss could therefore be the same in all chondrosarcomas. In similarity with hyperhaploid-hypodiploid ALL which commonly display loss of *CDKN2A* [13], all the present hyperhaploid-hypodiploid chondrosarcomas showed deletion of this gene. Previous reports have demonstrated lack of CDKN2A specifically in high-grade chondrosarcomas and suggested that this deficiency affects cell viability and proliferation [14], [15]. In the present study, all but one of the investigated tumors were of high grade. The majority displayed hyperhaploid-hypodiploid chromosome numbers or polyploidized clones with a hyperhaploid-hypodiploid origin. Many tumors displayed monosomy 9, or loss of chromosome arm 9p, with targeted deletion of *CDKN2A* in the remaining homologue resulting in complete absence of this gene. In this context it is interesting to note that lack of *CDKN2A* has been shown to induce supernumerary centrosomes through centriole pair splitting [16]. Cells that subsequently enter mitosis with more than two centrosomes may divide either through a pseudo-bipolar mitotic division or, more rarely, a multipolar mitotic division [16], [17], [18]. Both events can result in unequal segregation of genomic material. Cells reorienting from a multipolar to a bipolar state during metaphase-anaphase transition have been shown to survive despite having undergone chromosomal missegregation events, and are capable to again enter mitosis [17], [18]. Combined with selection for particular chromosomes, such cell divisions could create the hyperhaploid-hypodiploid clones detected in the present study. In cells affected by polyploidization not only the chromosomes but also the centrosomes have been duplicated. The disrupted chromosomal segregation observed in the current tumors following polyploidization may thus be attributed to acquired supernumerary centrosomes and subsequent pseudo-bipolar divisions [19], similar to the mechanisms by which polyploid hepatocytes recently have been shown to reduce their ploidy and generate aneuploid daughter cells [20].

In conclusion, we found that the majority of chondrosarcomas initially were affected by loss of chromosomes and that the pattern of lost and retained chromosomes was recurrent. Chromosomal loss was often followed by duplication of the entire genome. After polyploidization the cells may be affected by further gains, losses and rearrangements of genetic material, and even repeated rounds of polyploidization, creating highly complex karyotypes. Neither loss of chromosomes nor polyploidization was restricted to a particular chondrosarcoma subtype.

## Materials and Methods

### Ethics statement

All samples were obtained after informed consent and the study has been approved by the Lund University Ethics Review Board.

### Patient information and tumor samples

Tumors from 18 patients with skeletal chondrosarcoma, treated at the Departments of Orthopedics at the Skåne University Hospital or the Karolinska Hospital, Sweden, were included in the study. Sixteen of the tumors were selected for SNP array analysis based on previous array CGH findings showing an aberrant genomic profile which correlated well with results obtained by chromosome banding [7]. The correlation between cytogenetic and array CGH findings was used to ensure tumor representation of the DNA studied. The tumors were not selected based on the types of genomic aberrations present in the samples. Two tumors lacking suitable material for SNP array analysis were included for FISH analysis. The patients encompassed seven women and eleven men, with a median age of 69 years. Fourteen tumors were diagnosed as conventional and four as dedifferentiated chondrosarcoma. The tumors were located in the ribs (7), femur (5), tibia (1), foot (1), pelvis (1), scapula (1), sternum (1) and humerus (1). Based on radiographic appearance, twelve tumors were classified as central, one as peripheral, and five were of unclassifiable origin. The sizes of the tumors ranged from 4 to 20 cm; information was not available in one case. Using a three-grade scale, one tumor was classified as low-grade (grade 1) and the rest as high-grade (twelve grade 2 and five grade 3) chondrosarcomas according to the Evans grading scheme [21]. Follow-up ranged from 6 to 144 months (median 79 months). One patient was lost to follow-up. Of the remaining patients six developed metastases and three of these and two additional patients died from their disease. G-banding karyotypes were available in all cases. Detailed patient information can be found in Table S1.

### Cytogenetic data from the literature

To obtain further information on the frequency of haploidization and polyploidization in chondrosarcoma, as well as the distribution of modal chromosome numbers, we surveyed published karyotypes available in the Mitelman Database of Chromosome Aberrations and Gene Fusions in Cancer (2010) [5]. Ploidy levels were defined as described in ISCN 2009 [22].

### Whole genome DNA copy number and loss of heterozygosity analyses

SNP array analysis was used for combined DNA copy number and LOH investigation in cases 1–16. DNA previously extracted for array CGH was used for the present analysis. The DNA had been extracted from fresh frozen tumor biopsies using the DNeasy Tissue Kit including the optional RNaseH treatment, according to the manufacturer's instructions (Qiagen, Valencia, CA, USA). Quality and concentration of the extracted DNA were ascertained using a NanoDrop ND-1000 (Thermo Fisher Scientific Inc., Waltham, MA, USA). Tumor DNA was hybridized onto Illumina Human Omni-Quad v1.0 BeadChip or Human CNV370-Quad v3.0 BeadChip (Illumina, San Diego, CA, USA), containing 1.2 million and 370,000 markers, respectively, following standard protocols supplied by the manufacturer. Data were extracted from the GenomeStudio software (Illumina), and subsequently normalized and segmented using thresholded quantile normalization (tQN) and BAFsegmentation, respectively [23], [24]. Base pair positions are indicated according to the NCBI build 36 (hg18). SNP array data have been deposited in the Gene Expression Omnibus (GEO) database, www.ncbi.nlm.nih.gov/geo (accession no. GSE25985).

### Combined immunofluorescence and interphase fluorescence in situ hybridization analysis

The mouse monoclonal antibody pan Cadherin (ab22744, 5 µg/ml, Abcam, Cambridge, UK), detected by FITC-conjugated anti-mouse antibody (1∶100, Sigma-Aldrich, St. Louis, MO, USA), was used to visualize the plasma membrane of tumor cells in case 15, and in two additional chondrosarcomas without suitable material for SNP array analysis (cases 17 and 18) as well as in normal cartilage cells. The ploidy levels of the respective nuclei were determined by manually counting the number of centromeric probes specific for chromosomes known to be monosomic and disomic in hypodiploid cells and consequently disomic and tetrasomic in hypertriploid cells (Vysis, Downers Grove, IL, USA). In case 15, probes for centromeres 10 and 18 were used. In cases 17 and 18, as well as in normal cartilage, probes for centromeres 2 and 10 were used. IF and FISH analyses were performed as described with concurrent probe and target DNA denaturation at 85°C for 10 min [25].

## Supporting Information

Figure S1 **SNP array findings in cases 1–4.** Genomic loss, gain, uniparental disomy (UPD) and normal copy numbers are displayed for all autosomes in cases 1–4. Amplification denotes regions represented by more than four chromosomal copies. The imbalances maps were created using the freely available software Genome Wide Viewer (http://www.well.ox.ac.uk/~jcazier/GWA_View.html).
(TIF)

Figure S2 **SNP array findings in cases 5–8.** Genomic loss, gain, uniparental disomy (UPD) and normal copy numbers are displayed for all autosomes in cases 5–8. Amplification denotes regions represented by more than four chromosomal copies. The imbalances maps were created using the freely available software Genome Wide Viewer (http://www.well.ox.ac.uk/~jcazier/GWA_View.html).
(TIF)

Figure S3 **SNP array findings in cases 9–12.** Genomic loss, gain, uniparental disomy (UPD) and normal copy numbers are displayed for all autosomes in cases 9–12. Amplification denotes regions represented by more than four chromosomal copies. The imbalances maps were created using the freely available software Genome Wide Viewer (http://www.well.ox.ac.uk/~jcazier/GWA_View.html).
(TIF)

Figure S4 **SNP array findings in cases 13–16.** Genomic loss, gain, uniparental disomy (UPD) and normal copy numbers are displayed for all autosomes in cases 13–16. Amplification denotes regions represented by more than four chromosomal copies. The imbalances maps were created using the freely available software Genome Wide Viewer (http://www.well.ox.ac.uk/~jcazier/GWA_View.html).
(TIF)

Table S1 **Clinical and cytogenetic features.**
(DOC)

Table S2 **Ploidy levels of mono- and binucleated chondrosarcoma and cartilage cells detected by FISH.**
(DOC)
