# Correction: Clonal Evolution through Loss of Chromosomes and Subsequent Polyploidization in Chondrosarcoma

**DOI:** 10.1371/annotation/8f845569-8244-416b-b15e-89562177ce32

**Published:** 2011-10-10

**Authors:** Linda Olsson, Kajsa Paulsson, Judith V. M. G. Bovée, Karolin H. Nord

The complete author contributions are: Conceived and designed the experiments: KHN. Performed the experiments: LO, KHN. Analyzed the data: LO, KP, JVMGB, KHN. Wrote the paper: LO, KHN. 

