# Supplementary figures and images for: Clonal Evolution through Loss of Chromosomes and Subsequent Polyploidization in Chondrosarcoma
Source: PLoS One. 2011 Sep 20;6(9):e24977. doi: 10.1371/journal.pone.0024977 (PMC3176800; doi:10.1371/journal.pone.0024977)

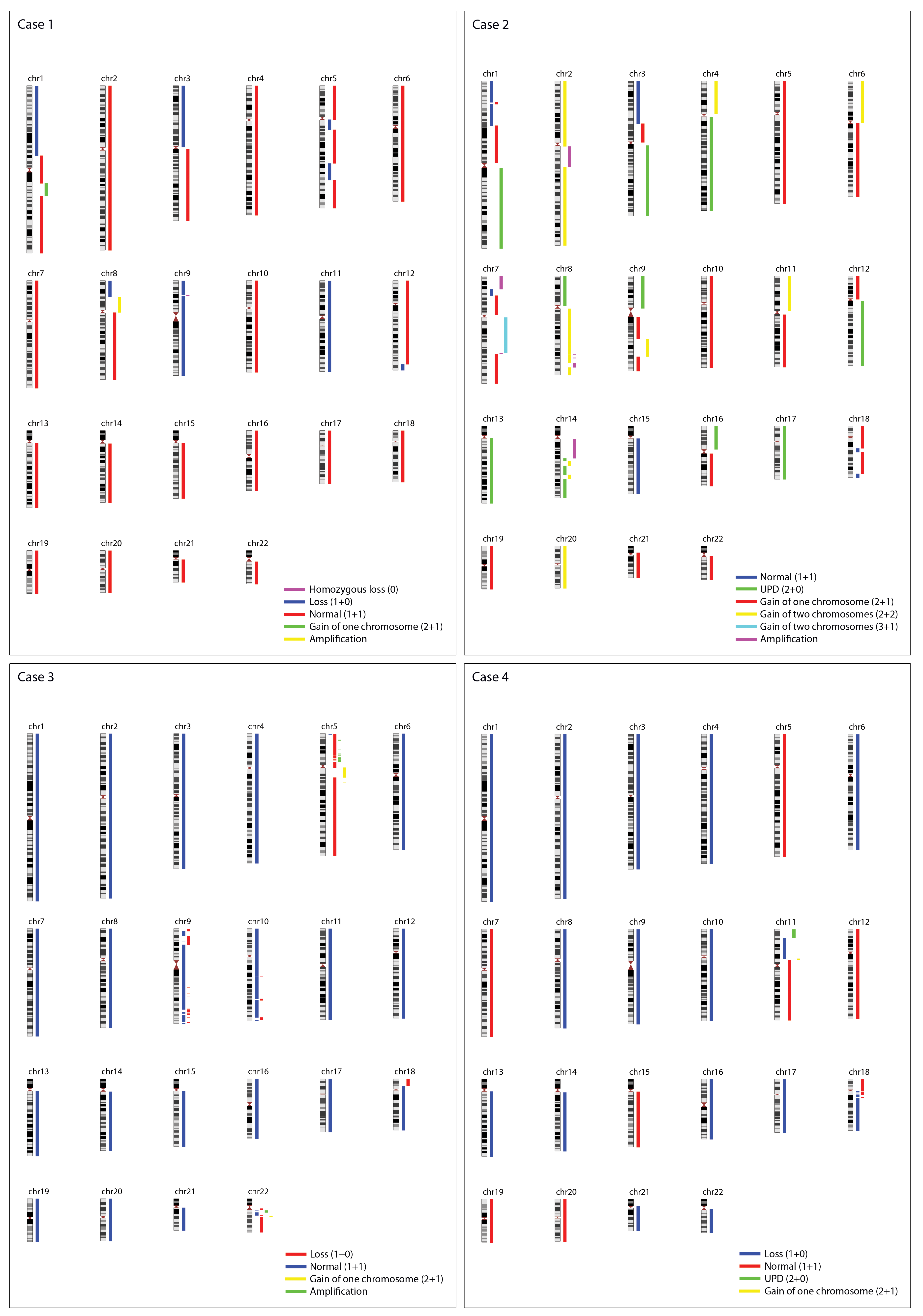

Supplement: Figure S1 — SNP array findings in cases 1–4. Genomic loss, gain, uniparental disomy (UPD) and normal copy numbers are displayed for all autosomes in cases 1–4. Amplification denotes regions represented by more than four chromosomal copies. The imbalances maps were created using the freely available software Genome Wide Viewer (http://www.well.ox.ac.uk/~jcazier/GWA_View.html). (TIF) [file pone.0024977.s001.tif]

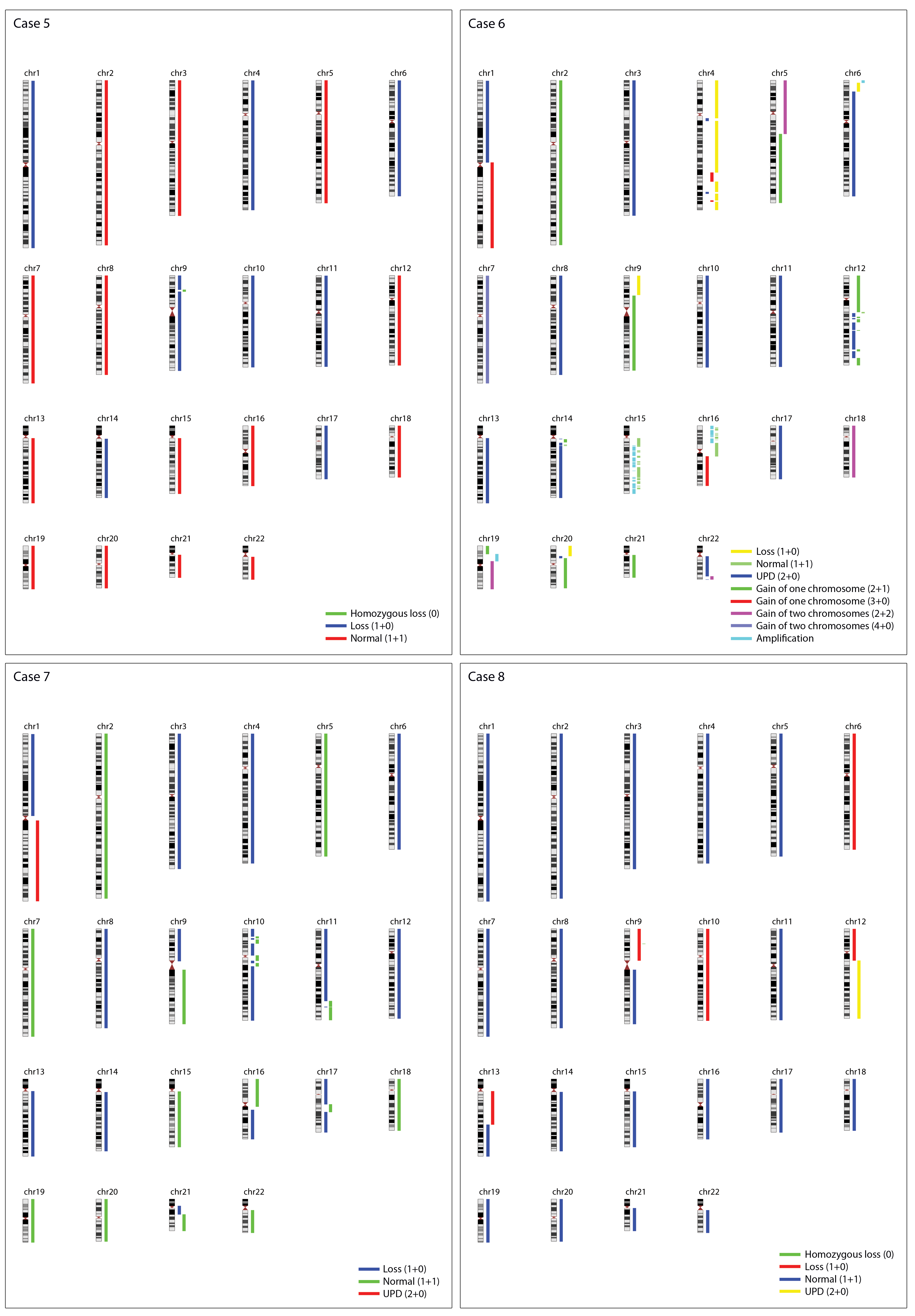

Supplement: Figure S2 — SNP array findings in cases 5–8. Genomic loss, gain, uniparental disomy (UPD) and normal copy numbers are displayed for all autosomes in cases 5–8. Amplification denotes regions represented by more than four chromosomal copies. The imbalances maps were created using the freely available software Genome Wide Viewer (http://www.well.ox.ac.uk/~jcazier/GWA_View.html). (TIF) [file pone.0024977.s002.tif]

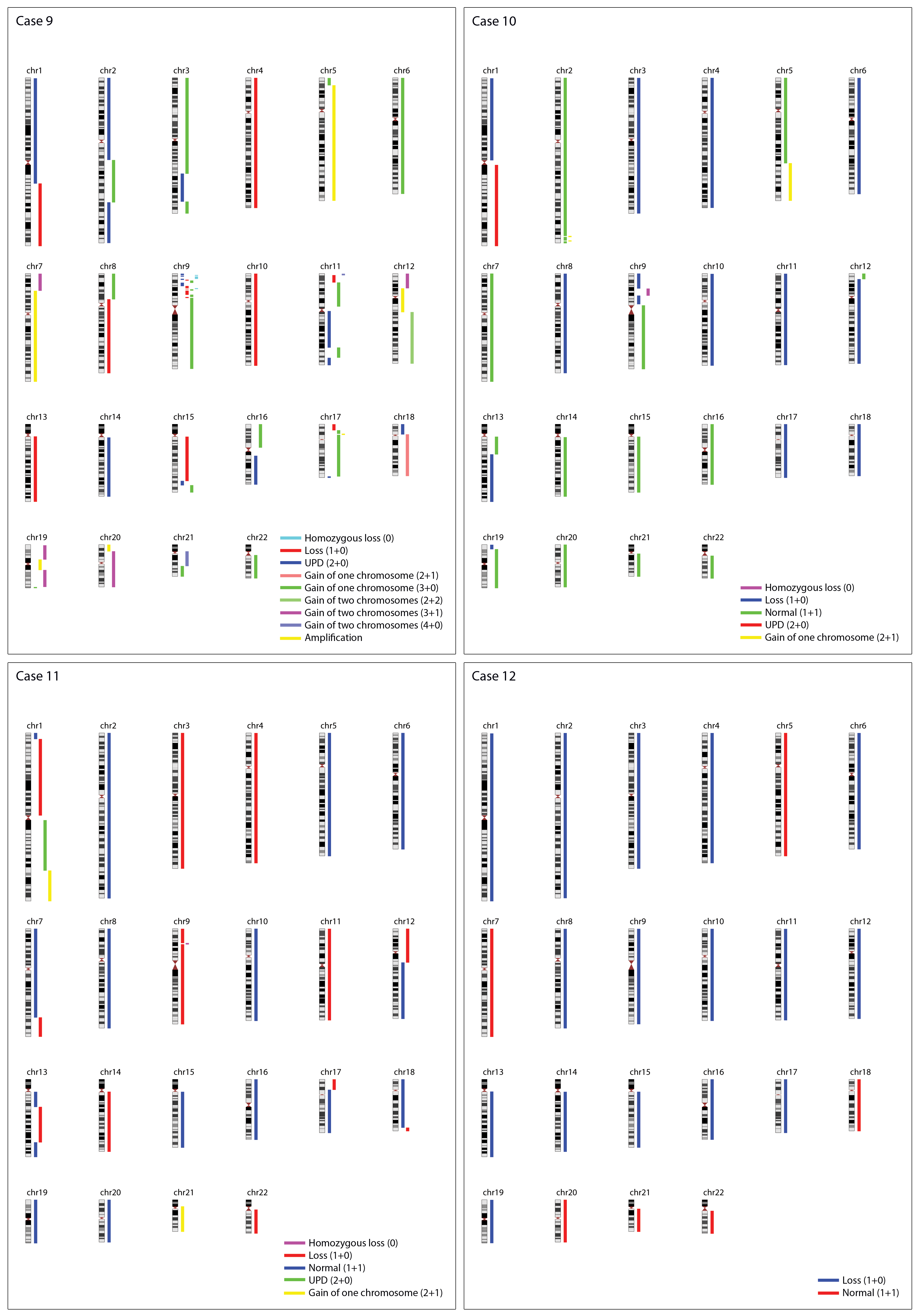

Supplement: Figure S3 — SNP array findings in cases 9–12. Genomic loss, gain, uniparental disomy (UPD) and normal copy numbers are displayed for all autosomes in cases 9–12. Amplification denotes regions represented by more than four chromosomal copies. The imbalances maps were created using the freely available software Genome Wide Viewer (http://www.well.ox.ac.uk/~jcazier/GWA_View.html). (TIF) [file pone.0024977.s003.tif]

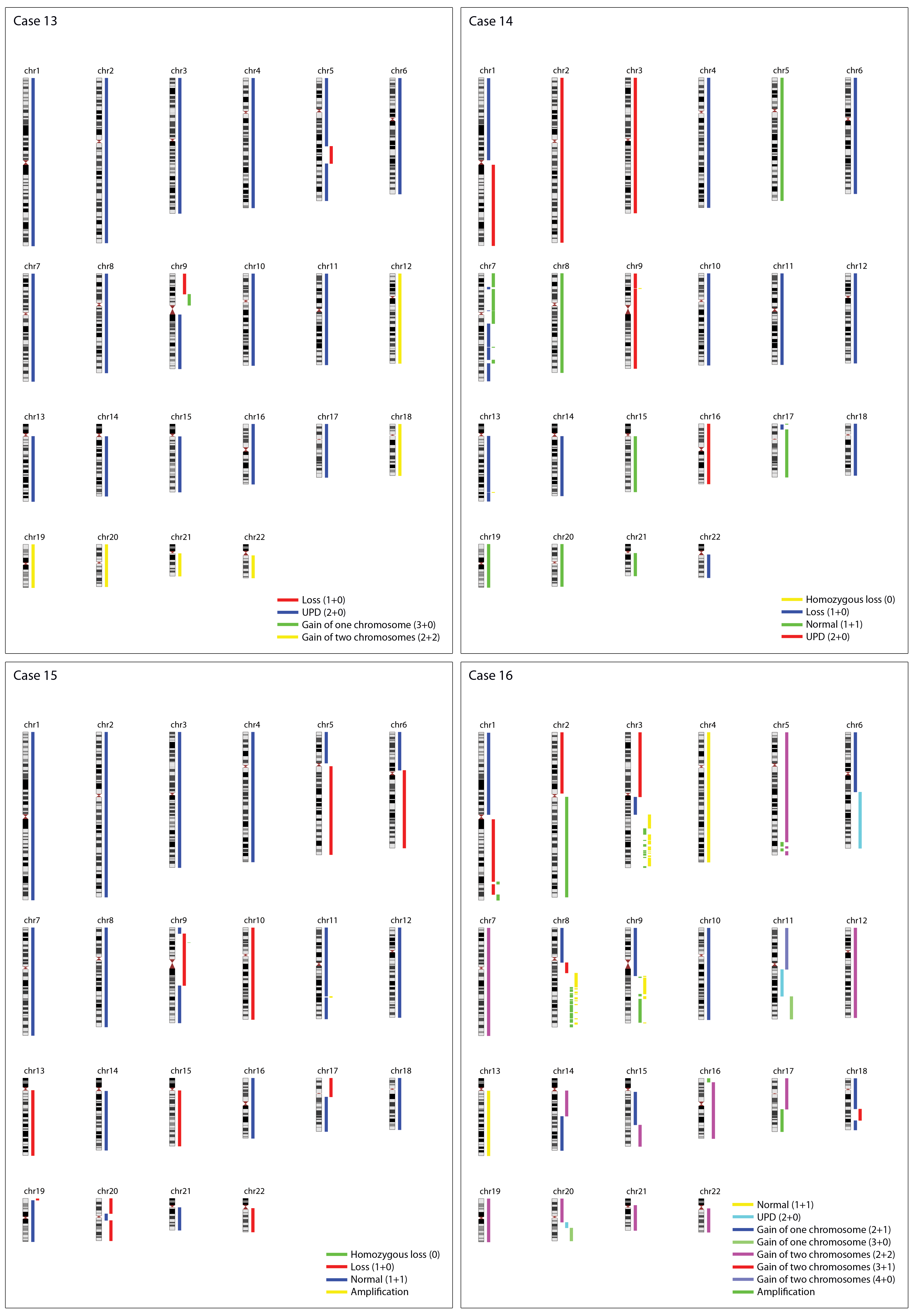

Supplement: Figure S4 — SNP array findings in cases 13–16. Genomic loss, gain, uniparental disomy (UPD) and normal copy numbers are displayed for all autosomes in cases 13–16. Amplification denotes regions represented by more than four chromosomal copies. The imbalances maps were created using the freely available software Genome Wide Viewer (http://www.well.ox.ac.uk/~jcazier/GWA_View.html). (TIF) [file pone.0024977.s004.tif]
