# Supplementary material for: Clonal Evolution through Loss of Chromosomes and Subsequent Polyploidization in Chondrosarcoma
Source: PLoS One. 2011 Sep 20;6(9):e24977. doi: 10.1371/journal.pone.0024977 (PMC3176800; doi:10.1371/journal.pone.0024977)
Supplement: Table S1 — Clinical and cytogenetic features. (DOC) [file pone.0024977.s005.doc]

Table S1. Clinical and cytogenetic features**a**.

| **Case^b^** | **Sex/**  **Age** | **Grade/**  **Type of surgery^c^** | **Site/Size**  **Follow-up^d^** | **Location^e^/**  **Type^f^** | **Karyotype^g^** | **Case no. in previous publication^h^** |
| --- | --- | --- | --- | --- | --- | --- |
| 1P | M/19 | 3/LEw | Femur/5  96 NED  R 15 | C/Conv | 43,XY,+X,-1,der(3;5)(q10;p10),-5,der(6)t(5;6)(q13;p21),add(8)(p11),-9,-11,der(12)t(6;12)(p21;q24), +r/42,idem,-Y/42,idem,der(2)t(2;7)(q13;q11),-4/41,XY,-1,der(3;5),-4,add(8),-9,-11,der(12)t(6;12),+r/ 42,XY,+X,-1,der(3;5)(q10;p10)ins(3;?)(q22;?),-5,-9,add(11)(p11),-12,der(12)t(6;12),-14,add(18) (q22),der(20)t(5;20)(q13;p21),+r | 9347:54  12869:1 |
| 2P | M/71 | 3/LEw | Femur/19  27 DoD  M 22 | C/Dediff | 62-63,add(X)(p22),-X,-Y,-1,add(3)(q12),-4,-5,+7,+del(7)(p11),+9,-10,-12,-13,-13,add(14)(q22),-16, -17,-18,der(18)t(16;18)(q11;q21),-19,+20,+21,der(21)t(9;21)(q11;p11)x1-2,+der(?)t(?;X)(?;q11), +der(?)t(?;13)(?;q14),dmin/103-109,XXX,add(X),add(3),add(4)(q35),ins(5;?)(q13;?),add(7)(p11), add(7)(p22),add(10)(q22),add(14),der(18)t(16;18),der(19)t(1;19)(p12;p13),der(21)t(9;21),+der(?) t(?;13),+1-2r,dmin,inc | 12869:2 |
| 3P^i^ | F/64 | 3/LEm | Pelvis/5  50 DoD  R 36 | C/Dediff | 44,XX,-5,dic(9;14)(p22;p13),add(10)(q26),add(18)(p11),+20,dic(20;20)(q13;q13),-22,+mar/75-86, idemx2,dic(14;14)(q32;q32) | 9347:4  12869:7 |
| 4P | M/50 | 1/LEm | Tibia/7  79 NED | C/Conv | 29,X,+5,+7,+der(11)t(11;15)(p1?3;q1?1),+12,+19,+20 | 9347:46  12869:9  Larramendy et al:6 |
| 5P | M/82 | 2/LEw | Rib/9  119 DoC | C/Conv | 63,XX,-Y,-1,-4,-6,-9,-10,+12,-17,-18,-19,+20,+22 | 9347:10  12869:14 |
| 6P | F/38 | 2/LEw | Rib/9  126 NED | C/Conv | 57-60,XX,del(1)(p?),+2,-4,-5,add(6)(p25),+7,+7,+8,add(9)(p24),+del(15)(q23),add(16)(p12), +19,der(20)t(1;20)(q21;q13)ins(20;?)(q13;?),+6mar | 9347:49  12869:19  Larramendy et al:7 |
| 7P | M/73 | 2/LEw | Rib/10  114 NED | C/Conv | 32,X,+2,+5,+7,+der(15)t(15;17)(p11;q21),+der(16)t(1;16)(q21;q13),+18,+19,+20,der(21)t(9;21) (q13;p11),+add(22)(p11) | 9347:51  12869:21 |
| 8P | M/50 | 2/LEw | Foot/4  136 NED  M 13, 48, 53 | C/Conv | 42,XY,-6,der(9;22)(q10;q10),-10,-12,del(13)(q?21),der(13)t(12;13)(q11;p11)/42,idem,del(11)(q23) | 9347:16  12869:26 |
| 9R | M/69 | 2/LEm | Femur/9  144 AwD  R 3, 60  M 72 | C/Conv | 75-79,+X,add(X)(q27)x2,-Y,der(1)t(1;2)(q32;q21),add(3)(q27),+der(3)t(3;14)(q21;q11)ins(3;?) (q21;?),-4,+5,+5,+5,+6,+7,+7,+7,-8,-9,-9,-10,der(10)t(5;10)(q21;p15)x2,add(11)(q25),+12,+12,+12, -13,der(13)t(2;13)(p11;p11),-14,add(14)(p?),-15,-15,+16,+17,add(17)(p13)x2,+18,+19,+19,add(19) (q13)x3,+20,add(20)(p13)x2,der(20)t(10;20)(q11;p13)x2,-21,-21,-21,inc | 9347:42  12869:34  Larramendy et al:44a+b |
| 10P | F/68 | 2/LEw | Rib/8  43 NED | C/Conv | 30-35,X,+2,+5,+7,+15,+der(19)t(9;19)(q13;p13),+der(20)t(1;20)(q?21;q13),+der(22)t(1;22) (q21;q13)/30-35,idem,del(1)(p?11) | 12869:35 |
| 11P | M/70 | 2/LEm | Rib/9  131 DoD  R 74, 94  M 94 | C/Conv | 38-40,XY,-1,t(2;5)(q37;q13),-3,-4,t(7;17)(q32;p13),-11,der(12)t(12;18)(q11;q23),-13,der(14)t(1;14) (q11;q13),der(18)t(12;18)(q13;q23),-22,+1-3mar/37-38,idem,-5,t(5;20)(p15;q11),-9,-10,-der(14) t(1;14),+der(14)add(14)(p11)t(1;14) | 9347:50  12869:36  Larramendy et al:19 |
| 12P | F/58 | 2/LEw | Humerus/8  48 NED | P/Conv | 27-29,X,+X,+5,+7,+18,+20,+21/54-59,idemx2,-X,dic(21;22)(p13;p13) | 12869:55 |
| 13P | M/79 | 2/LEw | Rib/10  51 DoC | NOS/Conv | 51-58,X,-Y,+12,+12,+18,+18,+19,+20,+20,+20,+21,+21,+22/54-56,XY,del(5)(q12q31),+12,+18, +19,+19,+20,+20,+21,+21/46,XY,t(1;12)(p36;q13) | 9347:57  12869:58 |
| 14P | F/64 | 2/LEm | Scapula/20  29 DoD  R 12, 15  M 27 | NOS/Conv | 35-37,XX,-1,-4,-6,-7,-10,-11,der(11)t(1;11)(q11;p15),-12,-13,add(13)(q34),-14,-18 | 9347:58  12869:59 |
| 15P | M/79 | 3/LE | Femur/?  LTF | NOS/Dediff | 70-77,XXY,+?del(X)(q22),+?Y,del(1)(p34),der(1)t(1;?11)(p36;q13)x2,+2,+3,-5,-6,+7,-9,der(9;15) (q10;q10),-10,+11,der(11)t(11;17)(q25;q11)x2,+12,-13,-14,der(14;22)(q10;q10),-15,-15,-17, ?add(17)(p11),+18,add(19)(p13)x2,?del(19)(p13),+?del(19),add(22)(q11)x2,+1-5r,inc | 12869:60 |
| 16R | F/21 | 2/LEw | Rib/4  136 NED  R 41  M 93 | NOS/Conv | 71-73,XXX,+2,+5,add(6)(q13-16),-10,-13,add(16)(p11),-17,-18,-19,?add(19)(q13),-20,+der(?)t(?;1) (?;q21)t(?;2)(?;q21),inc | 9347:40  12869:65 |
| 17P | M/70 | 2/None | Sternum/4  32 DoD | C/Conv | 39-40,X,-Y,del(1)(p22),add(4)(q35),-5,-6,add(6)(q2?2),add(9)(p13),-10,dic(11;21)(p11;p11),-14, -18,-20,?add(20)(p11),-22,+2-3mar/40-42,XY,-1,add(4),-6,add(7)(p22),add(9),-10,dic(11;21), add(16)(q21),-18,-22,+mar/74-81,XXY,-1,+2,+3,+4,add(4)x2,-6,add(9)x2,-10,+11,+11, dic(11;21)x2,+12,+15,+16,add(16)x2,+17,-18,+19,-20,+21,-22,inc/39-41,XY,dic(1;?) (p11;?),add(4),-5,-6,add(6),-9,-10,dic(11;21),-18,-20,-20,-22,+4mar/79-82,idemx2 | 12869:13 |
| 18P | F/80 | 3/LEm | Femur/14  6 NED | NOS/Dediff | 44,X,-X,del(6)(q22),del(9)(p2?2),-10 | - |

^a^ Data regarding patient age, tumor size, grade (three-grade scale), site and location, and surgical treatment concern the primary lesion only.

^b^ Source of material for cytogenetic and array analyses. P = primary tumor; R = local recurrence

^c^ LE = local excision with unknown, wide (LEw) or marginal (LEm) margins.

^d^ Follow-up time in months after diagnosis. DoC = dead of other causes; DoD = dead of disease; NED = no evidence of disease; AwD = alive with disease; LTF = lost to follow-up. Months to local recurrence (R) or metastasis (M) indicated when appropriate.

^e^ C = central; P = peripheral; NOS = unclassifiable.

^f^ Conv = conventional chondrosarcoma; Dediff = dedifferentiated chondrosarcoma.

^g^ Karyotypes are described according to the guidelines in ISCN (2009): An International System for Human Cytogenetic Nomenclature. Edited by Shaffer LG, Slovak ML, Campbell LJ. Basel, S. Karger, 2009.

^h^ Reference numbers are from the Mitelman Database of Chromosome Aberrations and Gene Fusions in Cancer (<http://cgap.nci.nih.gov/Chromosomes/Mitelman>). Larramendy ML, Mandahl N, Mertens F, Blomqvist C, Kivioia AH, et al. (1999) Clinical significance of genetic imbalances revealed by comparative genomic hybridization in chondrosarcomas. Hum Pathol 30: 1247-1253.

^i^ Radiotherapy and surgery for primary tumor. All other tumors were treated with surgery alone.
