# Supplementary material for: Clonal Evolution through Loss of Chromosomes and Subsequent Polyploidization in Chondrosarcoma
Source: PLoS One. 2011 Sep 20;6(9):e24977. doi: 10.1371/journal.pone.0024977 (PMC3176800; doi:10.1371/journal.pone.0024977)
Supplement: Table S2 — Ploidy levels of mono- and binucleated chondrosarcoma and cartilage cells detected by FISH. (DOC) [file pone.0024977.s006.doc]

Table S2. Ploidy levels of mono- and binucleated chondrosarcoma and cartilage cells detected by FISHa.

| **Case** | **Hypodiploid nuclei** | | **Hypertriploid nuclei** | | **Diploid nuclei** | |
| --- | --- | --- | --- | --- | --- | --- |
|  | **Mononuclear** | **Binuclear** | **Mononuclear** | **Binuclear** | **Mononuclear** | **Binuclear** |
| 15 | 20 | - (0%) | 144 | 10 (6%) | 750 | 10 (1%) |
| 17 | 92 | 1 (1%) | 5 | - (0%) | 250 | 29 (10%) |
| 18 | 362 | 39 (10%) | - | - | 106 | 1 (1%) |
| Control^b^ | - | - | - | - | 776 | 16 (2%) |

^a^ The number of detected cells are shown in the table and the percentages of binucleated cells of the respective ploidy levels are indicated within parentheses.

^b^ The control sample represents normal cartilage cells from three individuals.
